# Workload assessment of medical doctors at primary health care centers in the Duhok governorate

**DOI:** 10.1186/s12960-021-00664-2

**Published:** 2022-01-28

**Authors:** Samim Ahmed Al-Dabbagh, Hushyar Musa Sulaiman, Nazik Abdulrahman Abdulkarim

**Affiliations:** 1 Department of Family and Community Medicine, College of Medicine, University of Duhok, Duhok, Kurdistan Region Iraq; 2 Planning and Health Resource Development at the Duhok Directorate General of Health, Duhok, Kurdistan Region Iraq; 3 Division of Scientific Research, Department of Planning at the Duhok Directorate General of Health, Duhok, Kurdistan Region Iraq

**Keywords:** Workload, Physicians, Primary health care, WISN, Duhok

## Abstract

**Background:**

A shortage in human resources, particularly physicians, has become a challenge confronting health authorities in the Duhok governorate, as these resources are the key input for delivering health care. It has become necessary to identify the most appropriate scientifically sound method for having adequate staffing levels. This study aimed to forecast the required number of physicians to cope with the current workload at the main primary health care centers in the Duhok governorate.

**Methods:**

A cross-sectional study was adopted to collect data for 1 full year. Data collection included both primary and secondary data sources. A semi-structured questionnaire was developed to obtain information every month from health centers on activities related to training and leaves. Data analysis was performed using Workload Indicators of Staffing Need software.

**Results:**

Sixty-one primary health care centers met the final criteria for analysis. The study revealed physician shortages and inequity in the distribution of staffing. In these centers, 145 physicians lacked an adequate delivery of health services based on the workload imposed on them. The ‘workload indicators of staffing need’ ratio was 0.33, indicating high work pressure on medical doctors. Some centers offered more health care than others, but had fewer doctors based on the current staffing practices.

**Conclusions:**

This study pointed out the importance for the public health sector and academic medical institutions to use Workload Indicators of Staffing Needs software in health policy administration to restructure their efforts to address the physician shortages and distribution imbalances at primary health care facilities.

**Supplementary Information:**

The online version contains supplementary material available at 10.1186/s12960-021-00664-2.

## Background

A shortage of medical doctors at primary health care (PHC) facilities and the consequent workload pose a threat to equitably providing access to healthcare. The staff distribution system, particularly regarding physicians, currently in use is based on staff-to-population ratios and sometimes on fixed staffing patterns per type of health facility. Recent studies prove that a staff planning model focusing on the workload factor is essential in forecasting the required staff [1, 2].

In recent years, there has been a significant increase in attention paid to improving health service delivery outcomes at the primary, secondary, and tertiary levels globally. Since 2011, efforts have been made at the national and Kurdistan regional levels to modernize the health system in the country, targeting the six main blocks of the health system, including Human Resources for Health (HRH).

The World Health Organization (WHO) has recommended that countries need to ensure that the right people at the right number with the right skills in the right place at the right time and doing the right things are available to achieve universal health care coverage and, hence, social protection [1, 3, 4]. A shortage in HRH has been related to a mismatch between supply and demand and inequitable geographical distribution, and the migration of a skilled health workforce hinders the provision of equitable access to essential health services and, hence, health outcomes [5].

Calculating staffing requirements, including physicians, requires forecasting future needs in a specific facility or community to identify the optimal number of physicians for good functioning [2]. Any shortage in staff is associated with an increase in the workload of existing staff (burnout) and, ultimately, the potential risk of committing medical errors and compromising patient safety [6]. In recent decades, a range of models to forecast the right required staffing has evolved. Traditionally, HRH planning approaches have relied on applying current staff-to-population ratios to project future staffing requirements, such as doctors and nurses per 1000 population [1, 2]. However, this method falls short as the health needs of the population change over time and across geographical space.

This planning model does not consider the workload and productivity [1, 3, 7]. The Ministry of Health (MoH) proposed a standard fixed staffing level for each health facility level (health posts, primary health centers, district hospitals, etc.). However, this model is also not without shortcomings; it does not consider the work volume of different geographical areas [1, 2]. WHO has advocated shifting the direction toward the inclusion of workload in estimating staff requirements [4, 5]. With the first two approaches to staff estimation above, the health sector in the Duhok governorate has faced a significant challenge in deploying medical doctors most appropriately and equitably due to a shortage in the supply of medical doctors in general and the difference in their activities and, hence, their workload in particular. There is still a lack of surveys or studies on the latter approach in Iraq. To the best of our knowledge, the current study of workload assessment using the Workload Indicator of Staffing Needs (WISN) tool is the first of its kind in Iraq. The WISN is used for planning and managing human resources to improve the decision-making process at all levels of health services regarding priority setting to allocate/reallocate staff in a fair manner based on actual needs [2, 8, 9].

The current PHC services at the national, regional, and governorate levels are provided through different categories represented by PHC subcenters and main centers. A mix of medical and health workers, composed of physicians, nurses, dentists, physician assistants, public health staff, laboratory technicians, and other mid-level health professionals, plays their role in PHC. In main PHC centers, physicians play a significant role, particularly in medical consultation, antenatal care (ANC), postnatal care, family planning (FP), communicable disease notification, the management of malnutrition, breast cancer screening, and the management of hypertension and diabetes.

This study aims to evaluate the workload of physicians working at PHC centers in the Duhok governorate using the WISN tool. This study applies the WISN methodology to physicians’ workload in PHC centers, but recognizes the vital role of other types of health workers in PHC. More comprehensive WISN studies, including all professionals working in PHC teams, should be conducted in the future.

## Methods

The Duhok governorate is one of the four governorates of the Kurdistan region of Iraq, and it has a population of approximately 2.2 million, including a host community, internally displaced people, and refugees. This population is distributed over seven administrative districts: Duhok (650 741), Zakho (450 491), Summel (393 279), Akre (206 926), Sheikhan (170 629), Bardarash (158 113), and Amadiya (142 398) [10–12]. There are 61 main PHCs across these districts, ranging from 5 in the Sheikhan district to 18 in the Duhok district.

A descriptive cross-sectional study was conducted in these main PHC centers located in all administrative districts of the Duhok Governorate during a 1-year period. This study included only physicians working in public PHC centers.

Formal written consent was obtained from the Duhok Directorate General of Health (DGoH) to collect data relevant to nonworking days or absences and the training of all physicians working in PHC centers using a questionnaire designed for this purpose (see Additional file 1) and to access monthly workload data.

Official documents that define the expected workload components (preventive, curative, and administrative) taking up most of the physicians’ daily working time in PHC centers were reviewed and are listed in Table 1. Overall, 1 657 792 outpatient consultation visits, 12 620 first ANC visits, 25 029 s and subsequent ANC visits, 2063 first FP visits, 3078 s and subsequent FP visits and 8203 postnatal care visits were recorded. Three hundred ninety-six patients were treated for malnutrition, 38 678 were screened for hypertension and diabetes mellitus, and 2243 were screened for breast cancer. Additionally, 154 cases of communicable diseases were reported. The standard time used to deliver each activity listed in Table 1, identified through a desk review, was established using the expert judgment of selected medical doctors who had experience running family medicine facilities and preventive program managers at the Directorate of Preventive Health Affairs. Two types of activity standards were assessed: service standards and allowance standards. Service standards included activities that are reported in annual service statistics, e.g., outpatient consultations and deliveries, and were presented in terms of the unit of time. The standard workload is the amount of work (within one activity) that one person (physician) could perform in a year.

**Table 1:** Activity standard of physicians in PHC centers and 12-month workload

Physician activity	Time unit per patient (minutes)	Number of patients
ANC (first visit)	15	12 620
ANC (2nd plus visit)	5	25 029
FP (first visit)	20	2063
FP (2nd plus visit)	10	3078
Breast cancer screening	10	2243
Postnatal care	10	8203
Mild/moderate malnutrition	10	396
Screening for hypertension and diabetes mellitus	15	38 678
Notification for communicable diseases	15	154
Management and referral of cases	10	1 657 792

*ANC* antenatal care, *FP* family planning

Allowance standards are the activity standards of a staff category, which in our study is the physician, for support and additional activities that are not reported in annual services statistics. They can be expressed as an actual working time like other activity standards or be expressed by the percentage of working time. In this study, the working time (in minutes) was calculated automatically and added to other workload standards. There are two types of allowance standards: category and individual. Category allowance standards (CASs) included support activities that all the members of the involved category, i.e., in our study, physicians at PHC centers, share in performing. These included 3 h per week for health promotion and education and 2 h per month for meetings with other health staff in the center. Individual allowance standards (IASs) involved additional activities that require certain (a fixed number of) workers in a particular group to perform. In this study, two activities were included, for which the manager of the PHC center was the only responsible member. These included 1 h per week to check weekly reports of surveillance programs for communicable diseases issued to district managers and 1 h per week for checking and reviewing official administrative letters received from health directorates and districts.

The available working time (AWT) was estimated by reviewing the administrative logs of the Department of Administration at the Duhok DGoH to find the number of possible working days in the study year. This was calculated by multiplying the number of weeks in the year by the number of days a physician works in a week. The latter is estimated by combining the number of full-time and part-time working days. Furthermore, the number of days that the physicians were absent was documented. The documentation of absence days is done by reviewing the administration logs every month for public holidays announced by the government for public sector employees. Physicians were asked to complete the questionnaire every month during the study period and report monthly. Each PHC center was asked to report absence days, including annual leave, sick leave, training, and other leave. For each PHC, one report per the facility was used monthly. Once all these data were reported, the actual annual AWT was calculated by subtracting the sum of days off due to absence from the number of possible annual working days [3].

Actual annual workload data were also obtained prospectively from the Department of Planning and Directorate of Preventive Health, DGoH.

### Data analysis

The data generated from the different data sources were entered into WISN software, a human resource health planning tool developed by the WHO [1, 13]. The key input data entered into the WISN software related to AWT and workload statistics per activity for each PHC center [3].

Rounding the fraction of the calculated required staff was performed based on the principle stated by WHO (if necessary) [1].

Two main comparison results were derived from WISN analysis for a given PHC center. The first indicator was the difference between the actual and the required staff, which showed the level of surplus or shortage in physicians at PHC centers. The second indicator was the ratio of the actual to the required number of staff (WISN ratio). A WISN ratio = 1 indicates that the staff level was sufficient, a WISN ratio less than 1 indicates insufficient staff, and a WISN ratio greater than 1 indicates that the available physicians were more than enough.

## Results

### Available working time (AWT)

Physicians are entitled to weekly work represented by 5 full-time days from Saturday through Wednesday and one part-time day on Thursday. Full-time includes working 6 h in a day, while part-time is 5 h of work in a day. Thus, part-time days in the studied PHC meant approximately 0.85 days of the full-time day. It yielded an average of 5.85 working days in a week. There were 52 weeks in the year. Thus, the possible annual working days in the Duhok governorate were approximately 304.2 days. It was extracted by multiplying 5.85 possible working days in a week multiplied by 52 weeks that were available in the studied year. Table 2 indicates that the average number of days physicians were away from work other than the official weekends was 37 days. The actual AWT was calculated to be 267.2 (or approximately 267) days after subtracting 37 from 304.2.

**Table 2:** Days of physicians not in work at PHC centers of Duhok governorate during the study period

Reason for absence	Days absent
Public holidays	23
Annual leave	9
Sick leave	3
Other leaves (training, … etc.)	2
Total	37

This excludes official weekend days

### Shortage in/surplus of physicians at PHC centers in the Duhok governorate

Table 3 presents the required number of physicians to deal with the workload volume at PHC centers across the Duhok governorate. In general, in the eligible analyzed health centers, the Duhok governorate needed 145 more physicians than those available during the study time. The workload pressure was very high, with a WISN ratio of 0.33. Regarding the districts, the lowest WISN ratio was for Bardarash (0.22).

**Table 3:** Current and required number of physicians and WISN ratio per district, Duhok governorate

District	Current no. of physicians	Required physician no	Difference (current – required)	WISN ratio
Akre	7.00	17.00	− 10.00	0.48
Amadiya	16.00	28.00	− 12.00	0.50
Sumel	22.00	46.00	− 24.00	0.31
Zakho	16.00	58.00	− 42.00	0.26
Duhok	46.00	72.00	− 26.00	0.29
Bardarash	10.00	22.00	− 12.00	0.22
Sheikhan	10.00	29.00	− 19.00	0.28
Governorate average	127.00	272.00	− 145.00	0.33

Table 4 shows the number and percentage of health facilities with shortages in physicians based on the existing workload. Twenty percent of PHC centers had a shortage of 5 or more physicians, followed by 6% with a shortage of 4 physicians, 13% with a shortage of 3 physicians, 21% with a shortage of 2 physicians, and 18% with a shortage of one physician. Bardarash was the single district showing the most extreme shortage by the percentage of PHC centers (2 out of 4). Only 3 (5%) of the studied PHC centers had excess physicians based on workload, and 10 (16%) had the exact number of required physicians. The Zakho district was the only administrative district where all of its studied PHC centers had shortages in medical staffing.

**Table 4:** Number and percentage of health facilities with a shortage in physicians per current workload according to district

District	No. of studied PHCs	No. (%) of PHCs having surplus	No. (%) of PHCs with the exact number	No. (%) of PHCs with a shortage of				
				One physician	Two physicians	Three physicians	Four physicians	Five or more physicians
Akre	6	0	2 (33)	0	3 (50)	0	1 (16)	0
Amadiya	10	1 (10)	2 (20)	4 (40)	1 (10)	1 (10)	1 (10)	0
Bardarash	4	0	1 (25)	0	1 (25)	0	0	2 (50)
Duhok	18	2 (11)	3 (17)	3 (17)	5 (28)	4 (22)	0	1 (5)
Sheikhan	5	0	0	1 (20)	1 (20)	0	0	3 (60)
Sumel	8	0	2 (25)	1 (12)	0	1 (12)	1 (12)	3 (38)
Zakho	10	0	0	2 (20)	2 (20)	2 (20)	1 (10)	3 (30)
Governorate level	61	3 (5)	10 (16)	11 (18)	13 (21)	8 (13)	4 (6)	12 (20)

## Discussion

Ensuring and/or strengthening availability and access to health services is one of the pillars of efficient health systems and a fundamental approach to achieving universal health coverage (UHC) and Sustainable Development Goals (SDGs). SDG 3 calls for a substantial increase in the health workforce’s recruitment, development, training, and retention to ensure healthy lives and promote well-being for people of all ages [14]. Service provision is linked to inputs into the health system, including HRH [15].

This study was conducted to introduce a new approach to the health policy decision-making process at the level of the MoH in the Kurdistan region of Iraq in general and the level of the Duhok DGoH in particular. It contributed to a better understanding of shortages in medical staff by using WISN, an established analytical tool developed by the WHO, to incorporate workload data to estimate the required number of physicians more accurately.

The current study indicated that the Duhok governorate is critically short of physicians at PHC centers. An estimated shortage of 145 physicians compared to the already existing medical doctors in the studied PHC centers was found. An annual report by the Duhok DGoH in 2014 showed that the current density of main PHC centers was 0.5 main PHC centers per 10 000, which is far below the national target of one per 10 000 [16]. Increasing the number of main PHC centers to reach this target would further expand the deficit in primary care physicians and further delay the achievement of UHC.

The maldistribution of physicians across districts was evident. Although the Bardarash and Sheikhan districts had ten deployed physicians and five main PHCs per district, the Bardarash district had a lower WISN ratio (0.22 vs. 0.28), indicating a more significant shortage of physicians. This higher burden in the Bardarash district is partly explained by the unavailability of referral hospitals for PHCs and the remote location of the Bardarash district. In addition, the PHCs in this district have a higher number of visits by people for medical advice and other preventive programs.

Furthermore, Amadiya and Zakho had an average number of 16 physicians per district, but the WISN ratio of Zakho was much lower than that of Amadiya (0.28 vs. 0.50). In addition, all 10 PHCs in the Zakho district had a shortage of physicians, ranging from one in 2 facilities (20%) to 5 or more in 3 (30%) facilities. In contrast, none of the 10 PHCs in the Amadiya district had a shortage of 5 or more physicians, and 2 (20%) had an adequate number of physicians. The current norms of physician deployment to PHCs based on the number of physicians per health facility are misleading if the workload is not considered. Such standards may not always indicate an adequate supply per facility. They may overestimate or underestimate the actual needs.

The findings of this study raise interest in developing skill-mixing plans to shift some duties already assigned to medical doctors to other mid-level health care professionals. Skill-mixing substantially addresses shortages in specific staff, and when staff members in whom there is a shortage are substituted with a less expensive category of staff, the costs of providing of health services will be contained [17]. A systematic review also suggests that appropriately trained staff, particularly nurses, can deliver high-quality health care as physicians in primary care settings [18].

This study makes several contributions to workforce planning and management science compared to previous studies and surveys. A prospective survey was performed monthly to collect data on actual, not assumed, work activities. In addition, this work is representative of the governorate since all main PHC centers were included in the study with several exceptions. The threat to external validity could be minor.

Nevertheless, this study is not without shortcomings. Differences in the severity of illnesses for which patients sought consultation with medical doctors and subsequent differences in the time needed for physicians to perform a workup were not taken into account. Some patients may need more time from physicians, while others may need less. In this study, a 10-min cutoff was used for all. The findings of this study cannot be compared with previous studies [19] due to the variation in the conceptual framework of data collection and analysis among studies.

## Conclusions

The study has several policy implications for governmental health authorities. First, academic medical institutions and health authorities may need to restructure their plans to increase the supply of physicians based on community needs and the equitable distribution of graduated physicians. Second, shifting some current tasks to other mid-level health professionals such as nurses is also recommended. Third, the WISN tool can be considered an essential tool for use by health authorities to develop new norms for assessing the need for physicians at PHC centers and apply this tool to determine the distribution of physicians.

## Supplementary Information

**Additional file 1. **Kurdish questionnaire form—Daily work of physicians in primary health care centers in the Duhok governorate.

## Data Availability

The datasets used and analyzed during the current study are available from the corresponding author upon reasonable request.

## Abbreviations

ANC: Antenatal care
AWT: Available working time
CASs: Category allowance standards
DGoH: Directorate General of Health
HRH: Human Resources for Health
IASs: Individual allowance standards
MoH: Ministry of Health
PHC: Primary health care
FP: Family planning
WHO: World Health Organization
WISN: Workload Indicator of Staffing Needs
SDG: Sustainable Development Goals
UHC: Universal health coverage

## References

[1] Shipp PJ (1998). Workload indicators of staffing needs (WISN): a manual for implementation.

[2] Hossain B, Alam SA. Likely benefits of using workload indicators of staffing need (WISN) for human resources management and planning the health sector of Bangladesh. Hum Resour Dev J (HRDJ). 1999; 3(2):99–111. http://www.who.int/hrh/en/HRDJ_3_2_03.pdf. Accessed 14 Aug 2014.

[3] World Health Organization (2010). Workload indicators of staffing needs (WISN): user’s manual.

[4] Hagopian A, Manmth KM, Das A, Peter JH (2012). Applying WHO’s ‘workforce indicators of staffing needs’(WISN) method to calculate the health worker requirements for India’s maternal and child health services guarantees in Orissa State, Oxford University Press. Health Policy Plan J..

[5] Jimba M, Cometto G, Yamamoto T, Shiao L, Huicho L, Sheikh M. Health workforce: the critical pathway to universal health coverage. Background paper for the global symposium on health systems research, 16–19 Nov 2010, Montreux, Switzerland. http://healthsystemsresearch.org/hsr2010/images/stories/10health_workforce.pdf. Accessed 7 Dec 2014.

[6] Chen KY, Yang CM, Lien CH, Chiou HY, Lin MR, Chang HR (2013). Burnout, job satisfaction, and medical malpractice among physicians. Int J Med Sci.

[7] Ozcan S, Hornby P. Determining hospital workforce requirements: a case study. Hum Resour Dev J (HRDJ). 1999; 3(3): 210–20. https://www.who.int/hrh/en/HRDJ_3_3_05.pdf. Accessed 14 Aug 2014.

[8] Musau P, Nyongesa P, Shikhule A, Birech E, Kirui D, Njenga M (2008). Workload indicator of staffing need method in determining optimal staffing levels at Moi Teaching and Referral Hospital. East Afr Med J.

[9] Kolehmainen-Aitken R, Kromoredjo P, Mendr K, Darmawan J, Smith J (2009). A WISN Toolkit for implementing workload indicators of staffing need (WISN) to improve health workforce planning and management in decentralized heath system.

[10] Duhok Statistics Directorate. Letter No. 157 to: Duhok Directorate General of Health. 10 Feb 2015. 2 leaves. Duhok population 2009–2015.

[11] Duhok Directorate of Displacement and Migration. Letter No. 892 to: Duhok Directorate General of Health. 17 Feb 2015. 4 leaves. Internally displaced and migrated population in Duhok governorate in 2014.

[12] UNHCR. UNHCR registration trends for Syrian persons of concern as of 28-Feb-2015. https://data.unhcr.org/syrianrefugees/country.php?id=103. Accessed 13 Mar 2015.

[13] Workload indicators of staffing needs (WISN): software manual. Geneva: WHO; 2010.

[14] Buchan J, Dhillon IS, Campbell J (2017). Health employment and economic growth: an evidence base.

[15] WHO. Health service delivery. WHO; 2010. http://www.who.int/healthinfo/systems/WHO_MBHSS_2010_section1_web.pdf. Accessed 7 Dec 2014.

[16] Iraq MoH, WHO (2009). A basic health services package for Iraq.

[17] Dubois CA, Singh D (2009). From staff-mix to skill-mix and beyond: towards a systemic approach to health workforce management. Hum Resour Health.

[18] Laurant M, Reeves D, Hermens R, Braspenning J, Grol R, Sibbald B. Substitution of doctors by nurses in primary care. Cochrane Database Syst Rev. 2005;(2):CD001271. 10.1002/14651858.CD001271.pub2 15846614

[19] Daviaud E, Chopra M (2008). How much is not enough? Human resources requirements for primary health care: a case study from South Africa. Bull World Health Organ.

