# Supplementary material for: Workload assessment of medical doctors at primary health care centers in the Duhok governorate
Source: Hum Resour Health. 2022 Jan 28;19(Suppl 1):117. doi: 10.1186/s12960-021-00664-2 (PMC8796551; doi:10.1186/s12960-021-00664-2)
Supplement: Supplementary file 1 — Additional file 1. Kurdish questionnaire form—Daily work of physicians in primary health care centers in the Duhok governorate. [file 12960_2021_664_MOESM1_ESM.docx]

**Daily work of physicians in primary health care centers in Duhok governorate**

**Questionnaire form - Kurdish**

فورما كارىَ نوذداريَن بنطةهيَن ساخلةميىَ

ريَظةبةريا ساخلةميا/ كةرتىَ ....................

بنطةهىَ ساخلةميىَ ..........................

هةيظا ................ سالا ...............

هذمارا نوذداريَن بنطةهى ...............

- دةستويرييَن نوذدارا د ئةظىَ هةيظىَ:

| ناظىَ نوذدارى | هذمارا روذيَن دةستويردانىَ | ميَذويا دةستويريا |
| --- | --- | --- |
|  |  |  |
|  |  |  |
|  |  |  |

- خوليَن نوذدار لىَ ثشكدار بوى (سمينار, وورك شووث, كونفرانس يان هةر ضالاكيةكا زانستى):

| ناظىَ نوذدارى | ناظىَ خولىَ | ميَذويا دةستثيَكرن  وبدويماهيك هاتنا خولىَ | هذمارا دةمذميَريَن نوذدارى كار هيَلاى د هةر روذةكا خولىَ |
| --- | --- | --- | --- |
|  |  |  |  |
|  |  |  |  |
|  |  |  |  |

- كومبونيَن دناظ و دةرظةى بنكةهى ( لكةرتى, ناحيىَ, قائمقاميىَ ................) دظىَ هةيظىَ دا:

| **ناظىَ نوذدارى** | **ميَذو** | لايىَ كومبوون دطةل هاتية كرن | هذمارا دةمذميَريَن نوذدارى كار هيَلاى |
| --- | --- | --- | --- |
|  |  |  |  |
|  |  |  |  |
|  |  |  |  |

- سميناريَن رةوشةنبيريا ساخلةميىَ د ئةظىَ هةيظيَدا

| ناظىَ نوذدارى | ضالاكى | ميَذو | هذمارا دةمذميَرا |
| --- | --- | --- | --- |
|  |  |  |  |
|  |  |  |  |
|  |  |  |  |

- ضالاكييَن دى

| ناظىَ نوذدارى | ضالاكى | ميَذو | هذمارا دةمذميَرا |
| --- | --- | --- | --- |
|  |  |  |  |
|  |  |  |  |
|  |  |  |  |
